# Associations of prenatal maternal depressive symptoms with cord blood glucocorticoids and child hair cortisol levels in the project viva and the generation R cohorts: a prospective cohort study

**DOI:** 10.1186/s12887-023-04372-9

**Published:** 2023-10-28

**Authors:** Nathan J. Cohen, Serena Defina, Sheryl L. Rifas-Shiman, Sabrina Faleschini, Russell S. Kirby, Henian Chen, Ronee Wilson, Kimberly Fryer, Hanan El Marroun, Charlotte A.M. Cecil, Marie-France Hivert, Emily Oken, Henning Tiemeier, Amy C. Alman

**Affiliations:** 1 College of Public Health, University of South Florida, Tampa, FL USA; 2 Department of Child and Adolescent Psychiatry, Erasmus Medical Center, Rotterdam, The Netherlands; 3 Division of Chronic Disease Research Across the Lifecourse, Department of Population Medicine, Harvard Medical School, Harvard Pilgrim Health Care Institute, Boston, MA USA; 4 Department of Obstetrics and Gynecology, Morsani College of Medicine, University of South Florida, Tampa, FL USA; 5 Department of Pediatrics, Erasmus Medical Center, Rotterdam, The Netherlands; 6 Department of Psychology, Education, and Child Studies, Erasmus School of Social and Behavioral Sciences, Rotterdam, The Netherlands; 7 Department of Epidemiology, Erasmus Medical Center, Rotterdam, The Netherlands; 8 Molecular Epidemiology, Department of Biomedical Data Sciences, Leiden University Medical Center, Leiden, The Netherlands; 9 Diabetes Unit, Massachusetts General Hospital, Boston, MA USA; 10 Department of Nutrition, Harvard T.H. Chan School of Public Health, Boston, MA USA; 11 Department of Social and Behavioral Sciences, Harvard T.H. Chan School of Public Health, Boston, MA USA

**Keywords:** Depression, Glucocorticoids, Prenatal exposure delayed effects, Hair

## Abstract

**Background:**

Prior studies have reported conflicting results regarding the association of prenatal maternal depression with offspring cortisol levels. We examined associations of high levels of prenatal depressive symptoms with child cortisol biomarkers.

**Methods:**

In Project Viva (n = 925, Massachusetts USA), mothers reported their depressive symptoms using the Edinburgh Postnatal Depression Scale (EPDS) during pregnancy, cord blood glucocorticoids were measured at delivery, and child hair cortisol levels were measured in mid-childhood (mean (SD) age: 7.8 (0.8) years) and early adolescence (mean (SD) age: 13.2 (0.9) years). In the Generation R Study (n = 1644, Rotterdam, The Netherlands), mothers reported depressive symptoms using the Brief Symptom Inventory (BSI) during pregnancy, and child hair cortisol was measured at a mean (SD) age of 6.0 (0.5) years. We used cutoffs of ≥ 13 for the EPDS and > 0.75 for the BSI to indicate high levels of prenatal depressive symptoms. We used multivariable linear regression models adjusted for child sex and age (at outcome), and maternal pre-pregnancy BMI, education, social support from friends/family, pregnancy smoking status, marital status, and household income to assess associations separately in each cohort. We also meta-analyzed childhood hair cortisol results from both cohorts.

**Results:**

8.0% and 5.1% of women respectively experienced high levels of prenatal depressive symptoms in Project Viva and the Generation R Study. We found no associations between high levels of maternal depressive symptoms during pregnancy and child cortisol biomarkers in either cohort.

**Conclusions:**

The present study does not find support for the direct link between high levels of maternal depressive symptoms and offspring cortisol levels.

**Supplementary Information:**

The online version contains supplementary material available at 10.1186/s12887-023-04372-9.

## Background

Depression is a psychological condition characterized by symptoms that may include persistent sadness or a loss of interest in enjoyable activities [1]. In pregnancy, depressive symptoms are associated with an increased risk of delivering a low birthweight or preterm infant [2]. Maternal prenatal depressive symptoms may be associated with increased risks of developmental delay in infancy [3], internalizing and externalizing behaviors during childhood [4, 5], and social-emotional, cognitive, and motor development throughout childhood [6]. Other work has identified associations with maternal prenatal depressive symptoms and attention problems during childhood [7], and maternal prenatal diurnal cortisol levels are associated with worsened cognitive development during infancy, even after accounting for the effect of maternal prenatal depressive symptoms [8]. The fetal programming hypothesis [9], which proposes that adverse fetal exposures may predispose the infant to adverse health conditions later in life, provides a plausible explanation for the findings of all of these studies. Under this framework, some potential mechanisms include alterations of levels of maternal cytokines and reactive oxygen species [10]. In addition, prenatal stress may downregulate the expression of placental 11β-hydroxysteroid dehydrogenase type 2 (11β-HSD2) [11, 12], which protects the fetus from exposure to excess maternal cortisol [13].

Physiological perturbations associated with prenatal depressive symptoms may alter the placenta’s protective mechanisms that foster healthy fetal growth and development, potentially dysregulating the offspring’s hypothalamic pituitary adrenal (HPA) axis and resulting in abnormal offspring cortisol [14] and cortisone [15] levels. Prior research in this area has suggested that the dysregulation of the HPA axis could be attributed to epigenetic mechanisms programmed *in utero* [16]. It has also been suggested that fetal programming of the HPA axis may occur via downregulation of receptors that regulate the functionality of the offspring’s HPA axis or via decreased sensitivity of the receptors [17]. Additionally, it may also occur by reducing the densities of the offspring’s glucocorticoid receptors and mineralocorticoid receptors [18]. Furthermore, potential mediating factors, such as maternal cortisol levels [19] and birthweight [20] may play a role as well.

Previous studies of fetal programming of the HPA axis have showed contradictory results [21–27], with some studies identifying associations [24–26] and others finding no association [21–23, 27]. Few studies have assessed longer-term outcomes during childhood [25] and adolescence [24]. Furthermore, most prior studies have used saliva to measure cortisol [21–26], which can be used to assess the diurnal distribution of cortisol levels throughout the day [28]. Moreover, multiple studies have assessed associations between maternal prenatal depression and cortisol reactivity [29, 30], which refers to a spike in cortisol levels in response to a stressful event [31]. However, studies assessing baseline HPA axis activity, which refers to basal cortisol levels that experience natural diurnal variation when an individual is not experiencing a stressful event [32], are scarce. Furthermore, few studies have assessed cord blood cortisol [27] or hair cortisol as outcomes, which respectively have the advantage of assessing fetal HPA axis activity and chronic HPA axis activity during childhood.

The goal of this study was to assess associations of high levels of prenatal maternal depressive symptoms with cord blood glucocorticoids at birth and hair cortisol at mid-childhood and early adolescence in two prospective pre-birth cohorts. We hypothesized that high levels of prenatal depressive symptoms would be associated with higher levels of cortisol in exposed offspring at birth, mid-childhood, and early adolescence.

## Methods

### Study populations

The present study used data from two longitudinal pre-birth cohorts, Project Viva and the Generation R Study.

**Fig. 1:**
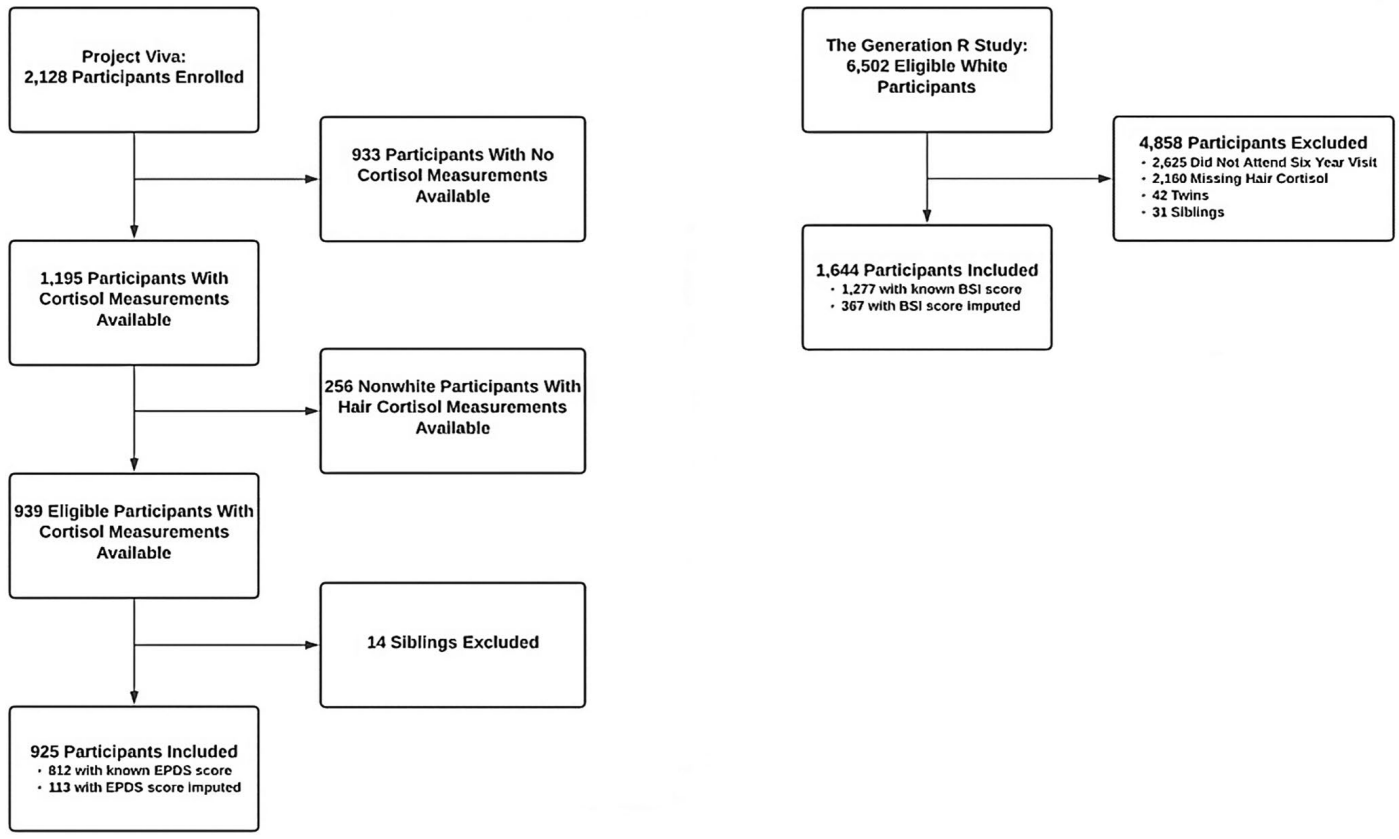
Flowchart of the Study Samples

Enrollment in Project Viva occurred from 1999 to 2002. We recruited women from obstetric clinics of Atrius Harvard Vanguard Medical Associates, a multispecialty group practice in eastern Massachusetts, USA, during their first prenatal visit. Inclusion criteria included bearing a singleton pregnancy, the ability to complete study questionnaires in English, the desire to continue residing in eastern Massachusetts after delivery, and being at ≤ 22 weeks of gestation at the time of enrollment. Of 2,128 live births, we excluded from the current analysis 1,189 children who did not have cortisol at any of the time-point assessments (Fig. 1). A small number of families included 2 sibling participants, thus we randomly excluded one sibling from each sibling pair using a random number generator in statistical software, which excluded an additional 14 children. Data from children of all races/ethnicities was included for the cord blood analyses, but due to the potential that variation in hair growth rates and textures by race/ethnicity may affect cortisol assays, only white children were included in the hair cortisol analyses [33]. Mothers reported on child race/ethnicity in early childhood, and we imputed missing values with maternal race/ethnicity. All mothers provided written informed consent, and the institutional review board of Harvard Pilgrim Health Care approved the study. We conducted the study in accordance with the guidelines from the Declaration of Helsinki.

Secondly, we used data from the Generation R Study. To be eligible for enrollment, women must have been expected to deliver between April 2002 and January 2006, and to have resided in Rotterdam, the Netherlands. The Generation R Study enrolled 6,502 eligible white participants, 3,877 of whom attended the study visit at six years of age. The present study excluded those with missing hair cortisol measurements (n = 2,160) in addition to twins (n = 42). Participants were missing hair cortisol measurements because they either did not visit the research center or were not approached for hair collection [34]. Hair collection began after the six-year study visits had already started, and it was offered universally to all participants thereafter [34]. We included only white participants in all hair cortisol analyses involving this cohort. Among sibling pairs, we randomly selected one sibling for inclusion in the present study, and we excluded the other one. This excluded an additional 31 children from the analyses. We included 1,644 children in the analyses (Fig. 1). The Medical Ethical Committee of the Erasmus Medical Center approved the study. We obtained written informed consent from all participants at enrollment, and we conducted the study in accordance with the ethical standards outlined in the Declaration of Helsinki.

### Prenatal maternal depressive symptoms

In Project Viva, mothers completed the Edinburgh Postnatal Depression Scale (EPDS) [35] at the mid-pregnancy visit (median 27.7 weeks gestation, IQR 26.6–28.7). The EPDS assesses depressive symptoms over the preceding seven days from when it is completed. The questionnaire has been validated for use during the pregnancy period [36]. The EPDS consists of 10 items, each of which is measured on a scale of 0–3 [35]. The EPDS is scored by summing up the responses to each of the 10 items; a higher score indicates greater levels of depressive symptoms. A score of ≥ 13 (on a scale of 0–30) indicates high levels of depressive symptoms [37]. The EPDS has been shown to have high validity for detecting clinical depression [35]. Cronbach’s alpha for the EPDS was 0.86 in our sample.

In the Generation R Study, mothers completed the Brief Symptom Inventory (BSI) [38] (median 13.2 weeks gestation, IQR 12.1–15.1). The BSI is a validated questionnaire that assesses symptoms of psychopathology over the past seven days. The BSI includes a subscale for assessing depressive symptoms, which has been shown to have strong agreement with the EPDS [39]. We calculated the total depressive symptom score from this questionnaire based on six items used to assess depressive symptoms [38]. The score on the survey ranges from 0 to 4 with a higher score denoting greater depressive symptoms. A score of > 0.75 indicates high levels of depressive symptoms [40]. For the depression subscale, Cronbach’s alpha was 0.80.

### Cord blood glucocorticoids

Cord blood glucocorticoids were measured only in Project Viva using umbilical cord venous blood at birth. The collection, processing, and storage of cord blood samples for the measurement of glucocorticoids in Project Viva have been described in detail elsewhere [41]. Briefly, cord blood samples were collected via syringe and needle from the umbilical vein by a hospital midwife or obstetrician. The collection of the cord blood was done carefully to prevent maternal contamination. The samples were refrigerated immediately after collection. The time of refrigeration depended on the time of delivery. Following refrigeration, the samples were separated into aliquots of serum, red blood cells, and white blood cells. A small validation study was conducted to confirm that refrigeration of the samples did not influence the glucocorticoid levels. Cord blood cortisol (MP Biomedicals, UK) and cortisone (Immunovation Ltd, Southampton, UK) were measured using validated radioimmunoassay techniques [42, 43] and radioimmunoassay kits for each glucocorticoid [44]. We calculated the ratio of cord blood cortisol to cortisone using the mean of duplicate cortisol and cortisone assays. The intra-assay coefficients of variation for cortisol were 5.6% and for cortisone 5.2%.

### Offspring hair cortisol

Offspring hair cortisol was measured at mid-childhood (mean (SD) age: 7.8 (0.8) years) and early adolescence (mean (SD) age: 13.2 (0.9) years) in Project Viva [45, 46], and at 6.0 (0.5) years of age in the Generation R Study [47]. Both studies used the same procedures for measuring hair cortisol. Research staff collected hair samples from the posterior vertex region of the scalp as close to the scalp as possible. Both cohorts assayed the proximal 3 cm of the collected hair to ensure that cortisol measurements reflected HPA axis activity over approximately the most recent three months. A laboratory staff member washed the hair sample with isopropanol, and then extracted cortisol using liquid chromatography tandem mass spectrometry [48]. The intra-assay coefficient of variation for hair cortisol was 9.6% in Project Viva, and the intra-individual coefficient of variation for hair cortisol was 14.0% in the Generation R Study.

### Other variables

We assessed self-reported demographic information at enrollment during pregnancy using questionnaires. We dichotomized maternal education as college graduate *yes*/*no*. Household income was dichotomized as an annual income >$40,000, *yes/no* for Project Viva and ≥ 1600€ per month for the Generation R Study, which is the approximate mean income for individuals residing in Rotterdam [49]. We dichotomized marital status based on whether the woman was married/cohabiting with her partner *yes*/*no*. We calculated maternal pre-pregnancy body mass index (BMI) based on height measured at enrollment and self-reported weight [50, 51]. We categorized smoking status into three groups: never smoked, former smoker, and smoker during pregnancy. We measured social support from friends/family on the early pregnancy questionnaire using five questions in Project Viva [52]. In the Generation R Study, we used the Family Assessment Device [53] to measure social support from family. We used the measure of social support from this questionnaire as a proxy for social support from friends/family.

### Statistical analysis

#### Statistical modeling

We performed univariable and multivariable linear regression models to examine associations of high levels of prenatal maternal depressive symptoms with cord blood glucocorticoids (in Project Viva) and with child hair cortisol (in both cohorts). We logarithmically transformed skewed outcome variables prior to analysis, including the hair cortisol outcomes. We standardized all continuous covariates and outcome variables (including the logarithmically transformed hair cortisol outcomes) that were included in the models using cohort specific internal z-scores.

For minimally adjusted models, we included child age (at hair collection)/gestational age (at delivery) and sex, plus race/ethnicity for cord blood outcomes. Hair cortisol analyses included only white children. For fully adjusted models, we additionally adjusted for maternal pre-pregnancy BMI, education, friend/family social support, pregnancy smoking status, marital status, and household income. We also fit a fully adjusted longitudinal model in Project Viva including both hair cortisol measurements to account for potential correlation between the two measurements. Additionally, we assessed effect modification by child sex via stratified analyses and tests for interaction.

#### Meta-analyses

We performed a meta-analysis using methods as described elsewhere [54] to examine the association between high levels of prenatal depressive symptoms and childhood hair cortisol levels across cohorts. We used a random effects model under the assumption that the effect size may differ between the Project Viva and Generation R cohorts. The meta-analyses involved combining hair cortisol measurements from the Generation R Study at a mean (SD) age of 6.0 (0.5) years with measurements taken at a mean (SD) age of 7.8 (0.8) years (in mid-childhood) in Project Viva.

#### Sensitivity analyses

We performed analyses excluding nonwhite participants for the cord blood outcomes in Project Viva to obtain a comparable sample to the hair cortisol analyses. We also conducted sensitivity analyses in both cohorts treating prenatal depressive symptoms as a continuous variable.

#### Missing Data

In all analyses, we used multiple imputation [55] to handle missing data on prenatal depressive symptoms and on all covariates, with the exception of child race/ethnicity. We did not impute child race/ethnicity in any analyses of hair cortisol due to the pre-specified requirement that analyses include only white children. Prior to imputation, there was a substantial amount of missing data in both cohorts. The approach to imputation included filling in missing values for prenatal depressive symptoms on a continuous scale.

We performed all meta-analyses and analyses of the Project Viva data in SAS (version 9.4), and we created all figures including the Project Viva data in R (version 3.6.1). We performed all analyses using data from the Generation R Study in R (version 4.0.5).

## Results

### Demographic characteristics of the study participants

**Fig. 2:**
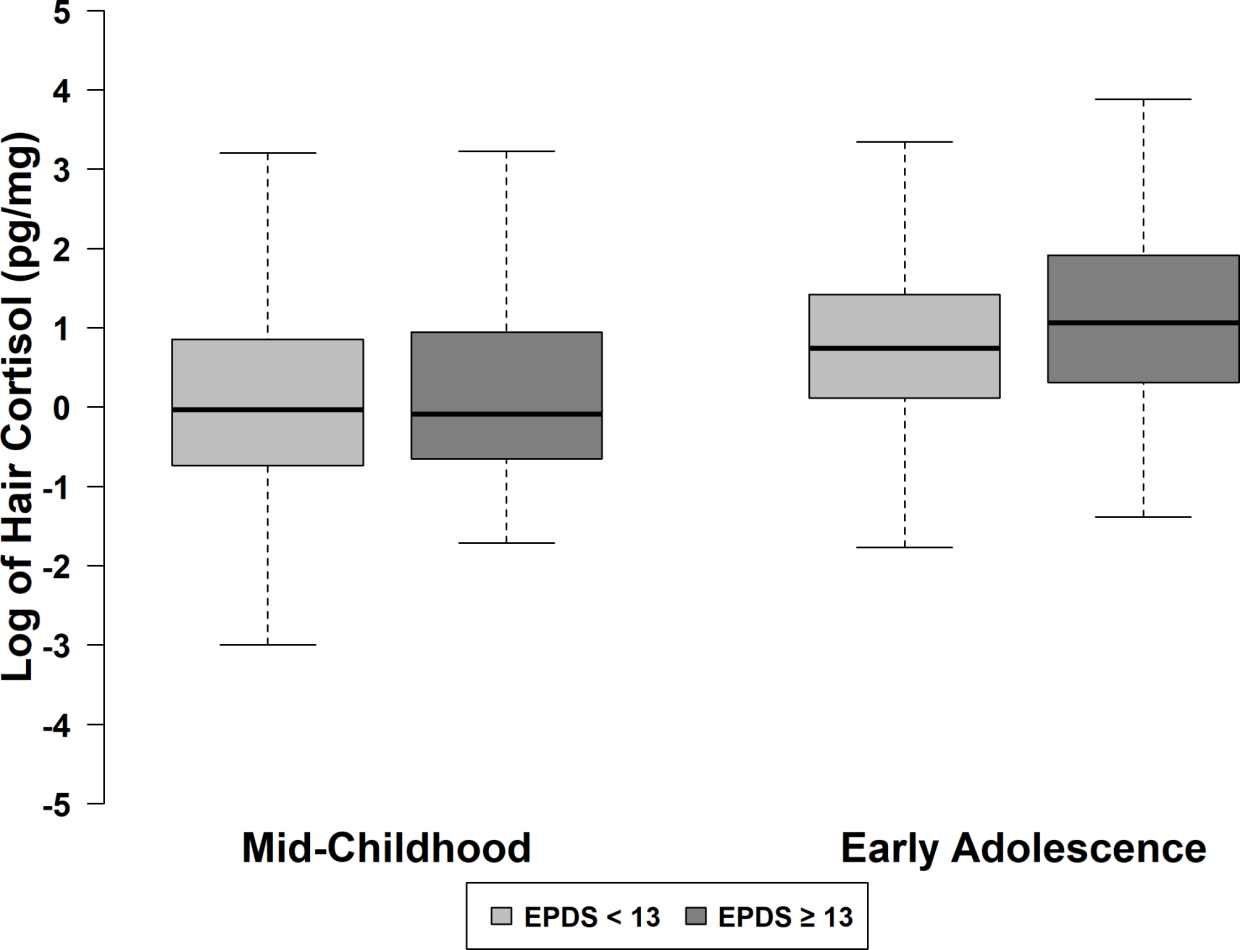
Distribution of Hair Cortisol in Project Viva by Levels of Prenatal Depressive Symptoms

Table 1 presents characteristics of Project Viva participants overall and according to level of prenatal depressive symptoms. Mean (SD) age at enrollment was 33.1 (4.5) years. In pregnancy, 65 (8.0%) women reported high levels of prenatal depressive symptoms. Among the included sample, 51.4% of the children were female. Women who experienced high levels of prenatal depressive symptoms were less likely to have a college degree or higher (70.8% vs. 78.6%) and more likely to smoke during pregnancy (12.3% vs. 7.5%), compared to women who did not. Additionally, a smaller percentage of women who experienced high levels of prenatal depressive symptoms were married or cohabitating with their partner (83.1% vs. 97.6%), and a smaller percentage had an annual household income >$40,000 (81.4% vs. 94.6%). At mid-childhood, median (IQR) hair cortisol was 0.97 (0.48, 2.35) pg/mg in children born to women who did not experience high levels of prenatal depressive symptoms and 0.92 (0.52, 2.57) pg/mg in children born to women who did. At early adolescence, median (IQR) hair cortisol was 2.10 (1.12, 4.15) and 2.89 (1.36, 6.80) pg/mg in each of these two respective groups (Fig. 2). The Spearman correlation between the two hair cortisol measurements was 0.13.

**Table 1:** Demographic Characteristics of Pregnant Women and their Children in Project Viva

	Overall^a^	EPDS < 13	EPDS ≥ 13
	n = 925	n = 747 (92%)	n = 65 (8%)
**Mother**			
Age at enrollment (years) (mean (SD))	33.1 (4.5)	33.2 (4.3)	31.8 (4.9)
Pre-pregnancy BMI (kg/m^2^) (mean (SD))	24.3 (4.8)	24.1 (4.7)	24.4 (5.0)
Education (N (%))			
Not a college graduate	214 (23.1)	160 (21.4)	19 (29.2)
College graduate	711 (76.9)	587 (78.6)	46 (70.8)
Pregnancy smoking status (N (%))			
Never	646 (70.1)	512 (68.8)	49 (75.4)
Former	202 (21.9)	176 (23.7)	8 (12.3)
Smoked during pregnancy	73 (7.9)	56 (7.5)	8 (12.3)
Marital Status (N (%))			
Not married or cohabiting	38 (4.1)	18 (2.4)	11 (16.9)
Married or cohabitating	887 (95.9)	729 (97.6)	54 (83.1)
Annual household income (N (%))			
≤$40,000	63 (7.1)	39 (5.4)	11 (18.6)
>$40,000	820 (92.9)	685 (94.6)	48 (81.4)
Friends/family social support (points) (mean (SD))	13.6 (2.1)	13.7 (2.0)	12.5 (3.0)
**Child at birth**			
Sex (N (%))			
Male	450 (48.6)	355 (47.5)	29 (44.6)
Female	475 (51.4)	392 (52.5)	36 (55.4)
Race/ethnicity (N (%))			
Black	45 (4.9)	27 (3.6)	10 (15.4)
Hispanic	22 (2.4)	13 (1.7)	2 (3.1)
Asian	11 (1.2)	9 (1.2)	2 (3.1)
White	805 (87.0)	667 (89.3)	49 (75.4)
More than one race/ethnicity	42 (4.5)	31 (4.1)	2 (3.1)
Gestational age (weeks) at birth (mean (SD))	39.7 (1.6)	39.7 (1.6)	39.5 (1.5)
Cord blood cortisol (nmol/L) (mean (SD)) [min, max]	340.3 (182.5) [33.1, 1076.0]	341.1 (190.4) [33.1, 1076.0]	355.1 (164.8) [137.9, 838.7]
Cord blood cortisone (nmol/L) (mean (SD)) [min, max]	241.4 (80.4) [56.0, 577.2]	240.2 (80.4) [56.0, 577.2]	251.6 (82.3) [110.5, 508.3]
Cortisol to cortisone ratio (units) (mean (SD)) [min, max]	1.43 (0.62) [0.15, 4.25]	1.43 (0.62) [0.15, 4.25]	1.52 (0.66) [0.50, 3.04]
**Child at mid-childhood**			
Age at mid-childhood hair cortisol measurement (years) (mean (SD))	7.8 (0.8)	7.8 (0.7)	7.9 (0.9)
Hair cortisol at mid-childhood (pg/mg) (median (IQR)) [min, max] ^b^	0.97 (0.50, 2.37) [0.01, 236.19]	0.97 (0.48, 2.35) [0.01, 236.19]	0.92 (0.52, 2.57) [0.01, 74.55]
**Child at early adolescence**			
Age at early adolescent hair cortisol measurement (years) (mean (SD))	13.2 (0.9)	13.2 (0.9)	13.1 (0.9)
Hair cortisol at early adolescence (pg/mg) (median (IQR)) [min, max] ^b^	2.19 (1.12, 4.42) [0.01, 200.96]	2.10 (1.12, 4.15) [0.01, 200.96]	2.89 (1.36, 6.80) [0.01, 48.69]
Change in hair cortisol from mid-childhood to early adolescence (pg/mg) (median (IQR)) [min, max] ^b^	0.75 (-0.51, 2.65) [-234.71, 194.70]	0.73 (-0.60, 2.63) [-234.71, 194.70]	0.98 (-0.13, 4.57) [-60.32, 44.80]

^a^ The “Overall” column includes participants with missing EPDS scores. For these participants, we imputed the missing EPDS score using multiple imputation in all analyses

^b^ Tabulations only include white children

**Fig. 3:**
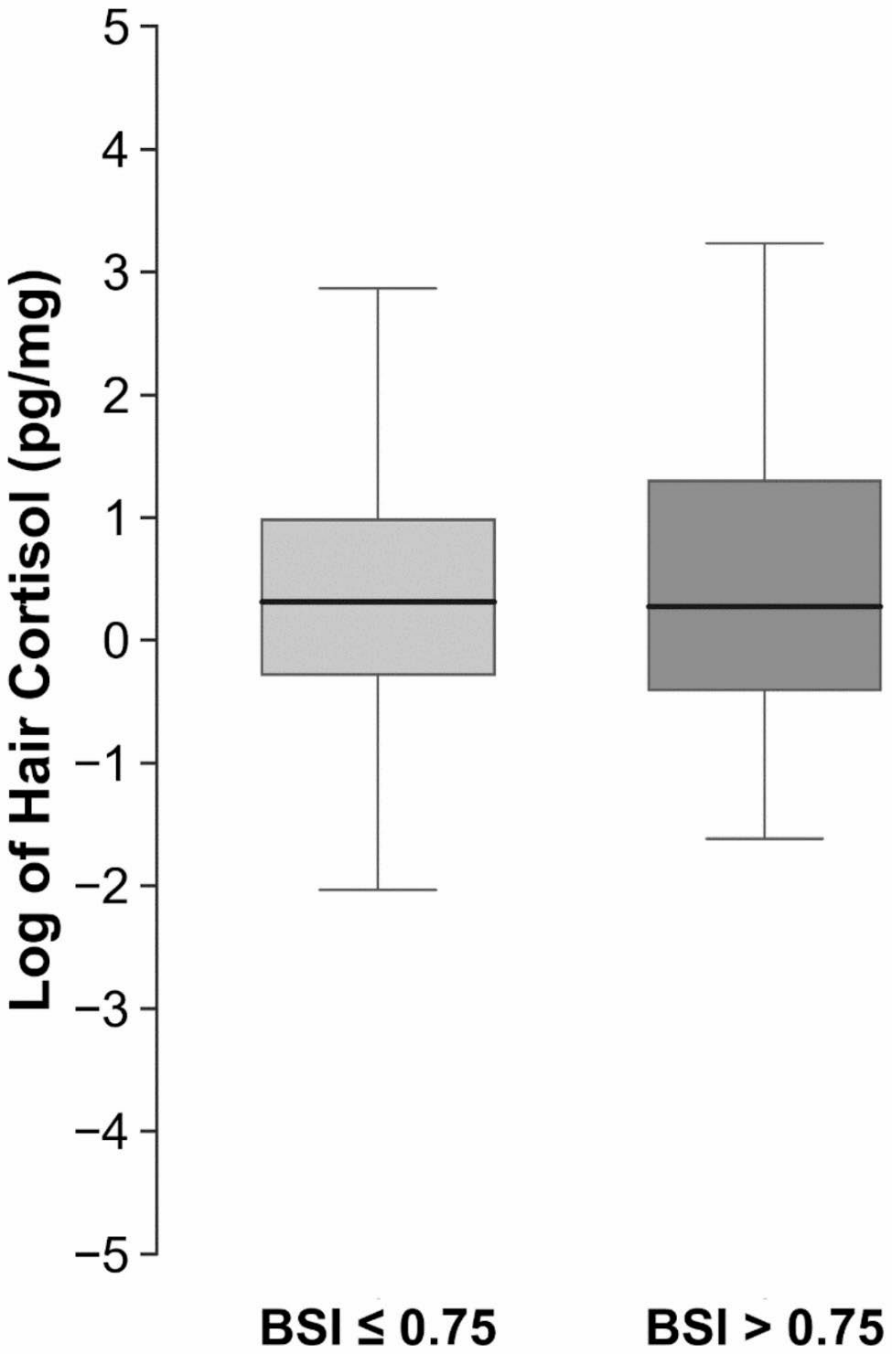
Distribution of Hair Cortisol in the Generation R Study by Levels of Prenatal Depressive Symptoms

Table 2 presents characteristics of the Generation R Study participants overall and according to the level of prenatal depressive symptoms. Mean (SD) maternal age at enrollment was 32.0 (4.3) years. In pregnancy, 65 (5.1%) women reported high levels of depressive symptoms. Half of the children in this cohort were female (50.3%). In this cohort, a smaller percentage of women who experienced high levels of prenatal depressive symptoms had a college degree or higher compared to those who did not experience them (45.9% vs. 65.7%). A greater percentage of women smoked during pregnancy among those who experienced high levels of prenatal depressive symptoms compared to those who did not (32.2% vs. 11.8%). Additionally, a smaller percentage of women who experienced high levels of prenatal depressive symptoms were married or cohabitating with their partner (76.2% vs. 95.8%), and a smaller percentage had an annual household income ≥ 1600€ per month (77.2% vs. 95.2%). The median (IQR) hair cortisol at age six was 1.36 (0.75, 2.66) pg/mg among children whose mothers did not experience high levels of prenatal depressive symptoms and 1.32 (0.67, 3.66) pg/mg among those whose mothers did (Fig. 3). A comparison of sociodemographic characteristics between the 1,644 children included in our analyses and the 6,502 eligible participants from the Generation R Study is shown in eTable 1. Mothers of children included in the current analyses were older, more educated, and less likely to smoke during pregnancy than mothers of children who were not included.

**Table 2:** Demographic Characteristics of Pregnant Women and their Children in the Generation R Study

	Overall^a^ n = 1644	BSI ≤ 0.75 n = 1212 (95%)	BSI > 0.75 n = 65 (5%)
**Mother**			
Age at enrollment (years) (mean (SD))	32.0 (4.3)	31.9 (4.1)	31.0 (5.2)
Pre-pregnancy BMI (kg/m^2^) (mean (SD))	23.2 (4.1)	23.2 (4.0)	23.1 (4.9)
Education (college graduate vs. not a college graduate) (N (%))			
No	572 (35.9)	412 (34.3)	33 (54.1)
Yes	1023 (64.1)	790 (65.7)	28 (45.9)
Pregnancy smoking status (N (%))			
Never	1165 (78.3)	873 (78.6)	32 (54.2)
Former	146 (9.8)	106 (9.5)	8 (13.6)
Smoked during pregnancy	177 (11.9)	131 (11.8)	19 (32.2)
Marital Status (N (%))			
Not married or cohabitating	82 (5.2)	50 (4.2)	15 (23.8)
Married or cohabitating	1496 (94.8)	1132 (95.8)	48 (76.2)
Annual household income (N (%))			
< 1600€/month (basic needs level)	81 (5.5)	53 (4.8)	13 (22.8)
≥ 1600€/month (basic needs level)	1392 (94.5)	1044 (95.2)	44 (77.2)
Social support (points) (mean (SD))	3.6 (0.4)	3.6 (0.4)	3.2 (0.5)
**Child at birth**			
Child sex (N (%))			
Male	817 (49.7)	585 (48.3)	37 (56.9)
Female	827 (50.3)	627 (51.7)	28 (43.1)
Gestational age (weeks) (mean (SD))	40.0 (1.8)	40.1 (1.6)	40.0 (2.2)
**Child at six-year study visit**			
Age at measurement of hair cortisol (years) (mean (SD))	6.0 (0.5)	6.0 (0.5)	6.0 (0.4)
Hair cortisol at age six (pg/mg) (median (IQR)) [min, max]	1.39 (0.76, 2.86) [0.13, 218.79]	1.36 (0.75, 2.66) [0.13, 159.01]	1.32 (0.67, 3.66) [0.20, 80.61]

^a^ The “Overall” column includes participants with missing BSI scores. For these participants, we imputed the missing BSI score using multiple imputation in all analyses

### Univariable and multivariable regression analyses

Table 3 presents results of the analyses between high levels of prenatal depressive symptoms and cord blood glucocorticoids in the Project Viva data. We did not observe any association in the unadjusted or the fully adjusted models for cortisol (β = 0.01 cohort specific internal z-scores, 95% CI -0.42, 0.45), cortisone (β = 0.10 cohort specific internal z-scores, 95% CI -0.33, 0.53) or the ratio of cord blood cortisol to cortisone (β = 0.12 cohort specific internal z-scores, 95% CI -0.32, 0.55).

**Table 3:** Associations of Maternal Prenatal Depressive Symptoms with Cord Blood Glucocorticoids and Child Hair Cortisol Levels^a^

	n	Project Viva Analyses		
		Unadjusted Model	Minimally Adjusted Model ^b^	Fully Adjusted Model ^c^
Cord blood cortisol	356	0.06 (-0.32, 0.44)	0.03 (-0.36, 0.42)	0.01 (-0.42, 0.45)
Cord blood cortisone	357	0.13 (-0.25, 0.52)	0.14 (-0.24, 0.55)	0.10 (-0.33, 0.53)
Cord blood cortisol/cortisone ratio	356	0.12 (-0.26, 0.50)	0.08 (-0.32, 0.47)	0.12 (-0.32, 0.55)
Hair cortisol at mid-childhood ^d^	628	0.13 (-0.20, 0.46)	0.15 (-0.18, 0.48)	0.13 (-0.21, 0.47)
Hair cortisol at early adolescence ^d^	556	0.19 (-0.16, 0.54)	0.19 (-0.15, 0.53)	0.18 (-0.16, 0.52)
Change in hair cortisol from mid-childhood to early adolescence ^d^	447	0.16 (-0.21, 0.54)	0.16 (-0.21, 0.53)	0.17 (-0.20, 0.55)
		**Generation R Analyses**		
	**n**	**Unadjusted Model**	**Minimally Adjusted Model** ^**b**^	**Fully Adjusted Model** ^**c**^
Hair cortisol at age six ^d^	1644	0.06 (-0.19, 0.31)	0.05 (-0.19, 0.30)	0.02 (-0.23, 0.26)
		**Meta-Analyses**		
	**n**	**Unadjusted Model**	**Minimally Adjusted Model** ^**b**^	**Fully Adjusted Model** ^**c**^
Hair cortisol at mid-childhood ^d^	2272	0.08 (-0.12, 0.28)	0.09 (-0.11, 0.28)	0.06 (-0.14, 0.26)

^a^ Reporting β (95% CI) comparing to the reference group, which is women without high levels of prenatal depressive symptoms. For all outcomes, the units are reported as cohort-specific internal z-scores

^b^ Adjusted for child age and sex. Cord blood outcomes additionally adjusted for child race/ethnicity

^c^ Minimally adjusted model additionally adjusted for maternal pre-pregnancy BMI, education, pregnancy smoking status, marital status, friend/family social support, and household income

^d^ Analyses only included white children

Table 3 also presents the results of the parallel analyses of hair cortisol conducted separately using data from each study and the meta-analyses of hair cortisol. We did not observe any associations in the fully adjusted models in the Generation R data at six years of age (β = 0.02 cohort specific internal z-scores, 95% CI -0.23, 0.26) and Project Viva data at mid-childhood (β = 0.13 cohort specific internal z-scores, 95% CI -0.21, 0.47). We also noted null associations in early adolescence in Project Viva (β = 0.18 cohort specific internal z-scores, 95% CI -0.16, 0.52). Consistent with these results, we observed no association in the longitudinal model for Project Viva (β = 0.15 cohort-specific internal z-scores, 95% CI -0.10, 0.39). Hair cortisol levels were similar between prenatally exposed and unexposed children in the fully adjusted meta-analysis (β = 0.06 cohort specific internal z-scores, 95% CI -0.14, 0.26).

In sensitivity analyses including only white children for the cord blood outcomes (eTable 2), we did not observe any association in unadjusted and fully adjusted models. Similarly, no associations were found using the continuous scores of the EPDS and the BSI as predictors in Project Viva and the Generation R Study. Lastly, we did not find evidence of effect modification by child sex in either study for any of the outcomes at birth, childhood, or early adolescence.

## Discussion

### Principal findings

This study examined the associations of high levels of prenatal maternal depressive symptoms with cord blood glucocorticoids and offspring hair cortisol levels in childhood and early adolescence in Project Viva and hair cortisol levels in the Generation R Study. In both studies, we did not observe any association between prenatal exposure to maternal depressive symptoms and cortisol biomarkers at any time point. These results suggest that high levels of maternal depressive symptoms may not alter levels of cord blood glucocorticoids or child hair cortisol.

### Interpretation

The theories of fetal programming and the developmental origins of health and disease [9] posit that adverse fetal exposures may lead to adverse health outcomes later in life. Specifically, fetal programming of the HPA axis may occur via dysregulation of glucocorticoid and mineralocorticoid receptors [17, 18]. Epigenetic mechanisms may also play a role, since maternal prenatal antidepressants have been associated with differential DNA methylation in the Project Viva and Generation R cohorts [56]. Additionally, prior research has implicated prenatal stress in the differential methylation of genes that regulate the functionality of the HPA axis [57]. However, a recent meta-analysis did not identify any associations between maternal prenatal anxiety and differential fetal DNA methylation in cord blood [58].

Our findings concur with previous work that found no association between high levels of prenatal depressive symptoms and child cortisol levels [22]. Our results also agree with a previous study conducted in the Generation R cohort, which found that maternal psychopathology more broadly was not associated with child hair cortisol [59]. However, that study found that maternal psychopathology, prenatal depressive symptoms, somatization, interpersonal sensitivity, anxiety, hostility, phobic anxiety, paranoid ideation, and psychoticism were all positively associated with child hair cortisone [59].

Our results run counter to prior research that identified a blunted salivary cortisol awakening response at age 15 in children who were prenatally exposed to high levels of maternal prenatal depressive symptoms [24] and other research identifying an association between high levels of maternal prenatal depressive symptoms and nighttime salivary cortisol levels at ages six to nine [25]. Our results are also not consistent with prior work that found an association between high levels of maternal prenatal depressive symptoms and infant salivary cortisol reactivity [26].

These inconsistencies across studies may be due to differences in the frequency and way in which cortisol was measured. In particular, the fact that hair cortisol measured in a 3 cm length of hair reflects long-term HPA axis activity over a period of about three months [60], whereas salivary cortisol measures acute HPA axis activity [61] may explain the discrepancies across studies.

Additionally, the discrepancies may be explained by differences in study size, confounding factors, and the socioeconomic compositions of the cohorts. For example, prior work has accounted for additional characteristics such as BMI during adolescence, smoking status during adolescence, and alcohol consumption during adolescence [24], but we did not adjust for these variables because they are post-exposure covariates that would likely be mediators, not confounders. Another prior study that identified an association included only four women with a university education [26], which could explain the discrepancies with the present study since we assessed two highly educated cohorts. Since our cohorts were highly educated, they likely included a smaller proportion of women with high levels of prenatal depressive symptoms than the general population. For example, previous research conducted among pregnant women born in Southwest England reported a prevalence of high levels of prenatal depressive symptoms of 17% and a prevalence of high levels of prenatal depressive symptoms of 25% in their daughters [62], both of which exceed the prevalence observed in either cohort in the present study. For comparison, the current study reported a prevalence of 8.0% in Project Viva and a prevalence of 5.1% in the Generation R Study. In addition, this previous study reported that < 50% of women in both generations had attained advanced level education qualifications [62], whereas college educated women comprised a majority of participants in both cohorts included in the current study.

### Strengths of the study

The present study has several strengths. Firstly, the inclusion of meta-analyses to obtain a robust estimate of the effect of high levels of maternal prenatal depressive symptoms on child hair cortisol levels is a strength, as it allowed us to obtain effect estimates pooled across both cohorts. Furthermore, the use of two data sources made our study more informative and allowed us to include two different cohorts that were enrolled in different clinical settings in our analyses, and the slightly different confounding structures of the two cohorts make our results more generalizable. Using hair cortisol as an outcome provided us with a robust and reliable physiological measure of chronic child HPA axis activation [60]. For this reason, we believe that the assessment of hair cortisol as an outcome fills a notable gap in the literature and adds to a body of literature that has primarily examined diurnal cortisol secretion and cortisol reactivity using saliva. Since most prior literature has examined salivary cortisol metrics, we also believe that our assessment of cord blood cortisol as a measure of fetal HPA axis activity could be seen as a strength.

### Limitations of the data

Despite its strengths, the present study has several limitations. Prenatal depressive symptoms were assessed in a self-reported questionnaire in Project Viva and the Generation R Study and depression was not clinically diagnosed by a physician. We also cannot generalize the results of the hair cortisol analyses to nonwhite racial groups given that we performed analyses only in white children due to differences in hair protein structure and cortisol storage making values incomparable. Additionally, we conducted our analyses in two socioeconomically advantaged cohorts, so our results cannot be generalized to more disadvantaged populations. Furthermore, depressive symptoms assessed at a single time point at mid-pregnancy may not represent the maternal mood throughout the entire duration of the pregnancy. However, we do not believe that levels of maternal depressive symptoms would considerably differ throughout the course of the pregnancy, especially given that prenatal depression is a strong risk factor for postpartum depression [63]. In addition, the small number of participants who experienced high levels of prenatal depressive symptoms in both cohorts may have resulted in low statistical power, which could be a potential explanation for our null findings. Furthermore, we did not consider potential moderating factors that could explain a potential link between high levels of prenatal depressive symptoms and child cortisol levels because we did not observe evidence for the association in our analysis. Our use of self-reported data on weight made our calculations of maternal pre-pregnancy BMI less reliable. Furthermore, we were unable to assess the cord blood outcomes in the Generation R Study because we did not have the data available. Finally, the use of cord blood and hair cortisol did not allow us to characterize diurnal variation in cortisol levels, which deserves further investigation by future research.

## Conclusions

Using data from two prospective pre-birth studies, Project Viva and the Generation R Study, we did not observe an association between high levels of prenatal maternal depressive symptoms and offspring cord blood corticosteroids at birth, and hair cortisol levels in childhood or adolescence. However, our results do not provide definitive evidence that a true association does not exist, so future research is needed in other cohorts to comprehensively answer the research question. Future studies would benefit from examining other perinatal conditions such as psychopathologies, stress or adverse postpartum environment in relation to offspring cortisol levels. Moreover, future research should also examine these relationships in a more racially/ethnically diverse sample using appropriate measures of HPA axis activation. Conducting research in these areas is an essential step in developing a comprehensive understanding of whether and how maternal prenatal depressive symptoms may affect long-term child health.

### Electronic supplementary material

Below is the link to the electronic supplementary material.

Supplementary Material 1

## Data Availability

The datasets generated and/or analysed during the current study are not publicly available due to public data sharing restrictions put in place to ensure participant privacy but are available from the corresponding author on reasonable request.

## List of Abbreviations

11β-HSD2: 11β-hydroxysteroid dehydrogenase type 2
HPA: Hypothalamic pituitary adrenal
EPDS: Edinburgh Postnatal Depression Scale
BSI: Brief Symptom Inventory
BMI: Body mass index
