# Supplementary material for: Associations of prenatal maternal depressive symptoms with cord blood glucocorticoids and child hair cortisol levels in the project viva and the generation R cohorts: a prospective cohort study
Source: BMC Pediatr. 2023 Oct 28;23:540. doi: 10.1186/s12887-023-04372-9 (PMC10612353; doi:10.1186/s12887-023-04372-9)
Supplement: Supplementary file 1 — Supplementary Material 1 [file 12887_2023_4372_MOESM1_ESM.docx]

eTable 1. Comparison of Demographic Characteristics of Pregnant Women and their Children in the Generation R Study Included in the Current Analyses to All Eligible White Participants

|  | **Included in Current Analyses**  **n=1644** | **All Eligible White Participants**  **n=6502** | **P-Value ^a^** |
| --- | --- | --- | --- |
| **Mother** |  |  |  |
| Age at enrollment (years) (mean (SD)) | 32.0 (4.3) | 31.6 (4.4) | 0.001 |
| Pre-pregnancy BMI (kg/m^2^) (mean (SD)) | 23.2 (4.1) | 23.1 (3.8) | 0.44 |
| Education (college graduate vs. not a college graduate) (N (%)) |  |  | 0.002 |
| No | 572 (35.9) | 1954 (40.2) |  |
| Yes | 1023 (64.1) | 2910 (59.8) |  |
| Pregnancy smoking status (N (%)) |  |  | 0.006 |
| Never | 1165 (78.3) | 3470 (75.4) |  |
| Former | 146 (9.8) | 430 (9.3) |  |
| Smoked during pregnancy | 177 (11.9) | 700 (15.2) |  |
| Marital Status (N (%)) |  |  | 0.27 |
| Not married or cohabitating | 82 (5.2) | 288 (6.0) |  |
| Married or cohabitating | 1496 (94.8) | 4529 (94.0) |  |
| Annual household income (N (%)) |  |  | >0.99 |
| < 1600€/month (basic needs level) | 81 (5.5) | 212 (5.5) |  |
| ≥ 1600€/month (basic needs level) | 1392 (94.5) | 3641 (94.5) |  |
| Social support (points) (mean (SD)) | 3.6 (0.4) | 3.5 (0.4) | <0.001 |
| **Child at birth** |  |  |  |
| Child sex (N (%)) |  |  | 0.67 |
| Male | 817 (49.7) | 2513 (50.4) |  |
| Female | 827 (50.3) | 2478 (49.6) |  |
| Gestational age (weeks) (mean (SD)) | 40.0 (1.8) | 39.8 (1.9) | <0.001 |

^a^ Based on a t-test for continuous variables and a chi-square test for categorical variables.

eTable 2. Sensitivity Analyses Including Only White Children for Cord Blood Glucocorticoids in Project Viva ^a^

|  | **Unadjusted Model** | **Minimally Adjusted Model ^b^** | **Fully Adjusted Model ^c^** |
| --- | --- | --- | --- |
| Cord blood cortisol | 0.07 (-0.50, 0.64) | 0.07 (-0.50, 0.65) | 0.01 (-0.60, 0.62) |
| Cord blood cortisone | 0.44 (-0.13, 1.01) | 0.44 (-0.12, 1.01) | 0.50 (-0.10, 1.11) |
| Cord blood cortisol/cortisone ratio | -0.04 (-0.62, 0.54) | -0.04 (-0.62, 0.54) | -0.14 (-0.75, 0.48) |

^a^ Reporting β (95% CI) comparing to the reference group, which is women without prenatal depressive symptoms. For all outcomes, the units are reported as cohort-specific internal z-scores.

^b^ Adjusted for child age and sex.

^c^ Minimally adjusted model additionally adjusted for maternal pre-pregnancy BMI, education, maternal household income, maternal social support, maternal smoking status, and maternal marital status.
